# Gene expression in bryozoan larvae suggest a fundamental importance of pre-patterned blastemic cells in the bryozoan life-cycle

**DOI:** 10.1186/2041-9139-2-13

**Published:** 2011-06-06

**Authors:** Judith Fuchs, Mark Q Martindale, Andreas Hejnol

**Affiliations:** 1 Department of Zoology, University of Gothenburg, Box 463, 40530 Göteborg, Sweden; 2 Kewalo Marine Laboratory, Pacific Biosciences Research Center, University of Hawaii, 41 Ahui Street, Honolulu, HI 96813, USA; 3 Sars International Centre for Marine Molecular Biology, University of Bergen, Thormøhlensgate 55, 5008 Bergen, Norway

## Abstract

**Background:**

Bryozoa is a clade of aquatic protostomes. The bryozoan life cycle typically comprises a larval stage, which metamorphoses into a sessile adult that proliferates by asexual budding to form colonies. The homology of bryozoan larvae with other protostome larvae is enigmatic. Bryozoan larvae exhibit blastemic tissues that contribute to build the adult during morphogenesis. However, it remains unclear if the cells of these tissues are pre-determined according to their future fate or if the cells are undifferentiated, pluripotent stem cells. Gene expression studies can help to identify molecular patterning of larval and adult tissues and enlighten the evolution of bryozoan life cycle stages.

**Results:**

We investigated the spatial expression of 13 developmental genes in the larval stage of the gymnolaemate bryozoan *Bugula neritina*. We found most genes expressed in discrete regions in larval blastemic tissues that form definitive components of the adult body plan. Only two of the 13 genes, *BnTropomyosin *and *BnFoxAB*, were exclusively expressed in larval tissues that are discarded during metamorphosis.

**Conclusions:**

Our results suggest that the larval blastemas in *Bugula *are pre-patterned according to their future fate in the adult. The gene expression patterns indicate that some of the bryozoan blastemas can be interpreted to correspond to homologous adult tissues of other animals. This study challenges an earlier proposed view that metazoan larvae share homologous undifferentiated "set-aside cells", and instead points to an independent origin of the bryozoan larval stage with respect to other lophotrochozoans.

## Background

Bryozoa (Ectoprocta) is a monophyletic group of sessile, colonial invertebrates and includes over 6,000 species in aquatic habitats worldwide [1]. Bryozoan life history, reproduction and anatomy are so fundamentally different from other metazoan groups (for example they lack typical circulatory structures or nephridia and the nervous systems of larvae and adults are unique), that traditional morphological investigations and the fossil record failed to clarify their evolutionary history. Bryozoa belong to Lophotrochozoa [2], but their phylogenetic position within the group is still ambiguous [3-6]. Recently, a few molecular studies indicated a close relationship of bryozoans with the clade Entoprocta + Cycliophora, but with low support [7,8]. Within Bryozoa, three major clades are recognized, Gymnolaemata (Eurystomata), Stenolaemata (Cyclostomata), and Phylactolaemata, but the phylogenetic interrelationships of these groups remain controversial [9-11].

Bryozoans have indirect development and their life cycle includes a sexually produced larval stage as well as asexual reproduction by cloning to give rise to colonial adult forms [12]. The most species rich bryozoan clade with over 5,000 species, the Gymnolaemata, has evolved a fascinating diversity of reproductive mechanisms and larval forms. Less than 20 species release their eggs directly into the surrounding water, where they develop into the well known cyphonautes larvae, planktotrophic (feeding) larvae with characteristic shells. Even fewer species produce shelled larvae with a non-functioning gut called pseudocyphonautes. The great majority of gymnolaemates, however, have evolved brood protection and their embryos develop into lecitotrophic (non-feeding) "coronate" larvae. Coronate larvae usually lack both a shell and a gut, but traces of a non-functioning gut were observed in some species, which was interpreted as a vestigial gut [13,14]. Despite the above outlined differences among gymnolaemate larvae, they usually share a set of morphological characters including transitory larval structures such as larval muscles and nerves (such as an apical organ), a glandulo-sensory organ (pyriform organ), internal cells, as well as blastemic cells that give rise to definitive adult tissues [14]. During a drastic metamorphosis, the transitory larval structures are discarded and only a single species is known to retain its larval gut in the adult [15]. Typically, all adult structures (for example gut, nervous system) are built *de novo *from the blastemic cells, which are found in various positions in the larvae and can give rise to different adult structures during metamorphosis in individual gymnolaemate species [16,17]. However, some authors propose that only ectodermal and mesodermal cells are involved in adult body plan formation from the larva, and this process mirrors the asexual budding process, which involves proliferation of the polypide (gut and lophophore) from the pluripotent body wall [12,14,18,19].

A comprehensive cell fate map does not exist for any species of bryozoan, so it is unknown if the blastemic cells in the larvae are undifferentiated, pluri- or multipotent set-aside/stem cells, or if these cells are determined for their future fate in the adult. To help to clarify the question to what extent the adult tissues are already determined in the larval stage, we investigated the spatial expression of 13 developmental genes in the newly released, coronate larvae of the gymnolaemate bryozoan *Bugula neritina*. This species is one of the better-studied bryozoans with respect to larval morphology and metamorphosis, although many details are still lacking. This initial set of genes was chosen according to the specific expression of genes in certain metazoan germ layers or organs as well as their putative conserved functions in the development among taxa, and represents a foundation for future molecular studies. Amongst the investigated genes, *Tropomyosin *is known to be a general marker of bilaterian musculature; the genes *Hox4, SoxB2, SoxE and FoxB *have functions in neural development; the genes *FoxA, GATA456 and Cdx (Caudal) *have been shown to be involved in gut patterning among taxa; expression of *GATA123 *and *BAMBI *was observed in the ectoderm of metazoans, and *Wnt *genes have been shown to be involved in multiple events including axial patterning (see Discussion). Genes engaged in bilaterian gut formation were investigated to determine if there is any molecular indication for a larval gut being present as anlage of the adult gut in *B. neritina*. We discuss the gene expression patterns of the bryozoan larval stage in the light of available data for other metazoans.

## Results

### Larval anatomy

**Figure 1:**
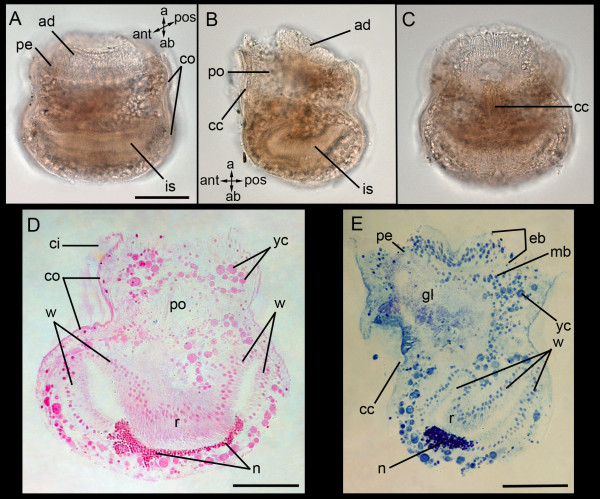
**Light micrographs showing the anatomy of *Bugula neritina *coronate larvae**. (**A**) Medio-anterior view, (**B**) lateral view, (**C**) anterior view. (**D**) Semithin section through a larva, posterio-median view; stain: basic fuchsine. (**E**) Semithin section of a larva, lateral view; stain: toluidine blue. a, apical; ab, abapical; ad, apical disc; ant, anterior; ci, cilia of coronal cells; cc, ciliated cleft; co, corona; ep, epidermal blastema; is, internal sac with wall (w), neck (n), and roof (r) regions; mb, mesodermal blastema; pe, pallial epithelium; po, pyriform (glandulo-sensory) organ; pos, posterior; yc, yolk inclusion. Scale bars (A) 100 μm, (D, E) 50 μm.

The larval anatomy of *Bugula neritina *has been previously studied and relevant data are reviewed here together with our own observations (Figure 1). The larvae have a roundish body shape (length, 260 μm; width, 190 μm) and the apical disc, which leads in swimming direction, marks the apical pole (Figure 1A, B). The apical disc comprises a central neural area (apical organ), which is surrounded by an outer epidermal blastema and an inner mesodermal blastema (Figure 1E) [14,20-22]. The apical organ is detected in semi-thin sections (not shown). The apical disc is encircled by infolded ectodermal cells, the pallial epithelium (Figure 1A, E). Opposite of the apical disc lies the large internal sac, which constitutes nearly half of the larval interior (Figure 1B). The internal sac is an ectodermal invagination used for attachment to the substrate during metamorphosis (see below) and comprises the neck, wall, and roof region [22]. The wall region is extensive and is folded upon itself (Figure 1D, E). The roof cells are elongated and contain basal inclusions of electron transparent vesicles [22]. The neck cells are recognized by their dark inclusions (Figure 1D, E). Here, we define the region of the internal sac opening as "abapical" instead of the formerly used "oral", since coronate larvae lack a mouth. The anterior side of the larva is defined by the ciliated cleft and the pyriform organ, a typical gymnolaemate larval organ (Figure 1C, D). It is a glandular organ, used for crawling, sensing, and transitorily attaching the larva to the substrate prior to metamorphosis [14]. The pyriform organ extends between the mesodermal blastema and the internal sac (Figure 1B, E). The larva possesses additional nerves, muscles, and yolky cells (Figure 1D) [12,20,23]. Some of the internal (yolky) cells are probably endodermal and mesodermal [14]; however, neither their origin nor their contribution to the adult body plan is resolved. Most of the larval surface is covered by several hundred coronal cells, which constitute the larval swimming organ (Figure 1A, D) [14].

The terminology that describes the blastemic cell layers in bryozoans is currently confusing and it will be useful to change the nomenclature for the blastemic cells in the future, when comprehensive cell lineage studies become established, which trace the embryological origin of these cells. Herein, we define the cells of the internal sac, the pallial epithelium, the epidermal blastema, and the mesodermal blastema as blastemic cells.

### Development

**Figure 2:**
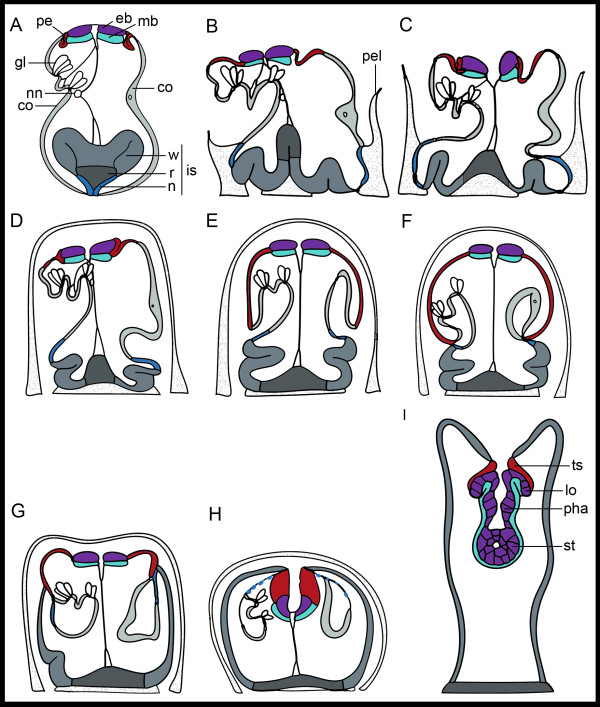
**Schematics of the fate of larval structures during the metamorphosis of *Bugula neritina***. Descriptions and slightly modified drawings after [21,22,76]. **A**) Competent larva. **B**) Five seconds (s): post settlement: Initial attachment through eversion of the internal sac. **C**) 20 s: The roof region of the internal sac moves towards the substratum, the apical disc retracts. **D**) 60 s: The apical disc re-extends, the corona starts involuting, the pellicle (excreted by the neck region) covers the larva. **E**) Approximately 120 s: The corona involutes, the pallial epithelium covers the apical hemisphere, the wall rotates. **F**) Approximately 160 s: The edge of the pallial epithelium constricts and the apical disc is compressed, bringing the pallial epithelium into contact with neck and wall of the internal sac. **G**) Approximately 240 s. The pallial epithelium constricts and the wall rises towards the apical region. **H**) 360 to 390 s. The pallial epithelium thickens, the wall covers the apical region, the apical disc and surrounding pallial epithelium are pulled inside. Coronal cells begin to autolyse. **I**) Several hours: transformation from the preancestrula to ancestrula: the pre-ancestrula elongates, the wall region forms the epidermis (which then secretes the cuticle and calcium carbonate). Invaginated cells of the epidermal blastema form the digestive tract and the lophophore. The splanchnic lining is formed by cells of the mesodermal blastema. The pallial epithelium has formed the tentacle sheath. co, corona (light grey); eb, epidermal blastema (purple); gl, gland cells of pyriform organ; lo, developing lophophore; mb, mesodermal blastema (cyan); nn, nerve nodule; pe, pallial epithelium; pha, pharynx; pel, pellicle, secreted by the neck region; st, stomach; ts, tentacle sheath; internal sac (is) consisting of n, neck (blue); r, roof (dark grey); and w, wall (grey).

All data outlined in this section and in Figure 2 were gathered from previous studies. *Bugula neritina *is a brooder and embryos are nourished by a special placenta-like system [18]. The free swimming period of the larva is short, probably between one and 30 hours before settlement [24]. Initial attachment of the larva to the substrate and first morphogenetic movements take only a few minutes and the metamorphosis to the first feeding adult is accomplished within several hours (Figure 2A-I) [18,21]. The blastemic tissue of the internal sac aids in attachment of the larva to the substrate. The neck region of the internal sac contains dark inclusions aiding in initial attachment before being discarded, while the roof region contains secretory material and forms the permanent attachment disc of the first individual of the colony (ancestrula) [16,19]. The wall region of the internal sac subsequently forms the epidermis of the free surface of the ancestrula [19]. The epidermal blastema forms the digestive tract and the lophophore of the adult bryozoan [25]. The mesodermal blastema contributes the lophophoral coelomic lining and the splanchnic peritoneum, while the origin of the somatic peritoneum is unclear [22]. The pallial epithelium forms the epithelium of the tentacle sheath of the adult. Transitory larval structures such as the apical organ, the pyriform organ, and the corona are discarded at metamorphosis.

### Gene expression patterns in the bryozoan larval stage

Here we describe the spatial expression of 13 developmental genes in the larval stage of *Bugula neritina *according to gene families.

#### Tropomyosin

**Figure 3:**
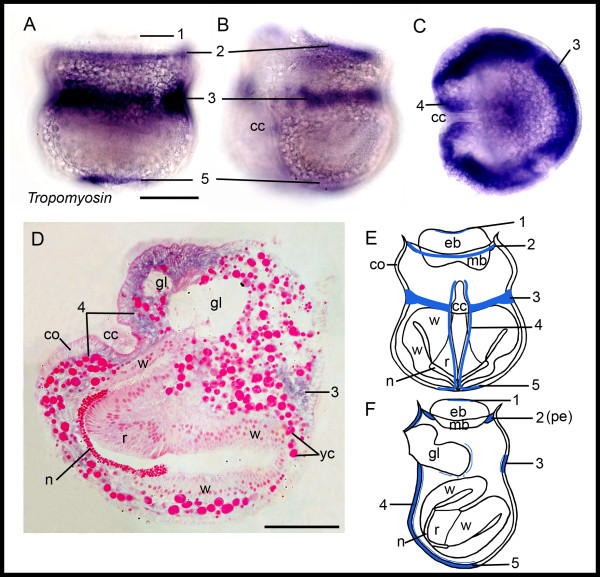
***Tropomyosin *expression in *Bugula neritina *larvae**. Expression apical (1), at the pallial epithelium (2), at the equator (3), along the ciliated cleft (4), in the abapical region (5). (**A-C**) Light micrographs of whole-mounted larvae. Larvae in posterior (A), lateral (B), and abapical view at the equator (C). (**D**) Light micrograph of semithin section of larva in lateral view showing the expression in coronal cells as well as cells underneath. (**E, F**) Schematics of *BnTropomyosin *expression. (E) Larva in posterior/median view, (F) Larva in lateral view. cc, ciliated cleft; co, coronal cells, eb, epidermal blastema; gl, gland cells of pyriform organ; mb, mesodermal blastema; n, neck; pe, pallial epithelium; r, roof; w, wall; yc, yolk inclusions. Scale bars (A) 100 μm, (D) 50 μm.

In the larvae of *Bugula neritina*, we found *BnTropomyosin *expression at the upper edge of the apical disc, along the pallial epithelium, surrounding the larva in an equatorial position, as well as alongside the ciliated cleft and internally around the glandular area of the pyriform organ (Figure 3A-F). Additional expression is found in the abapical region below the internal sac (Figure 3A, B). Overall, *BnTropomyosin *expression is strongest in the coronal cells at the larval surface as well as in cells just below the coronal cells, suggesting the presence of myoepithelial cells in these areas (Figure 3D).

#### Hox4

**Figure 4:**
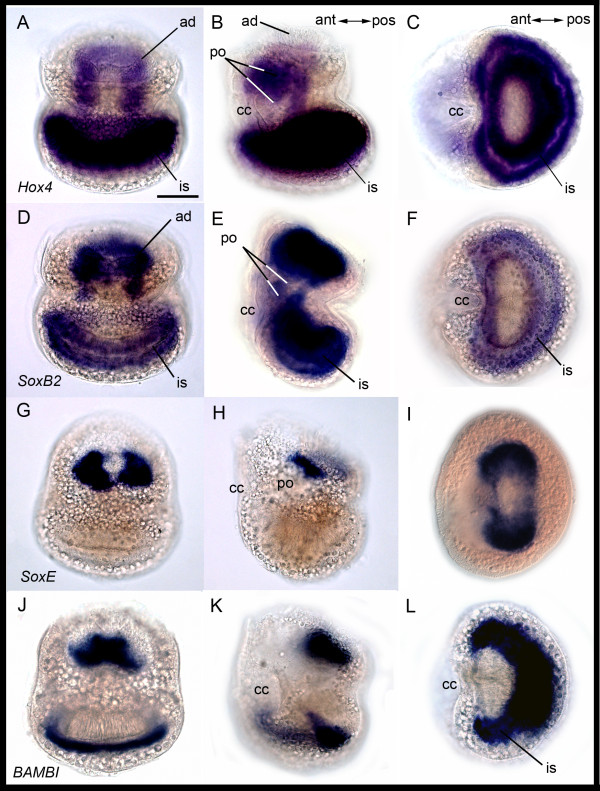
**Gene expression in *Bugula neritina *larvae**. Column 1: Posterior view. Column 2: Lateral view, with anterior (cc = ciliated cleft) to the left. Column 3: Abapical view except I, apical view. Anterior pointing left. Row 1: *BnHox4 *expression in the apical disc, the glandular complex (pyriform organ = po), and parts of the internal sac. Row 2: *BnSoxB2 *expression similar to *BnHox4 *expression. Row 3: *BnSoxE *expression in the mesodermal blastema. Row 4: *BnBAMBI *expression in a trapezoid area including epidermal, mesodermal, and internal cells. Expression is also found in a horseshoe shaped area in the internal sac corresponding to some wall cells. ad, apical disc; ant, anterior; is, internal sac; pos, posterior. Scale bar 100 μm.

**Figure 5:**
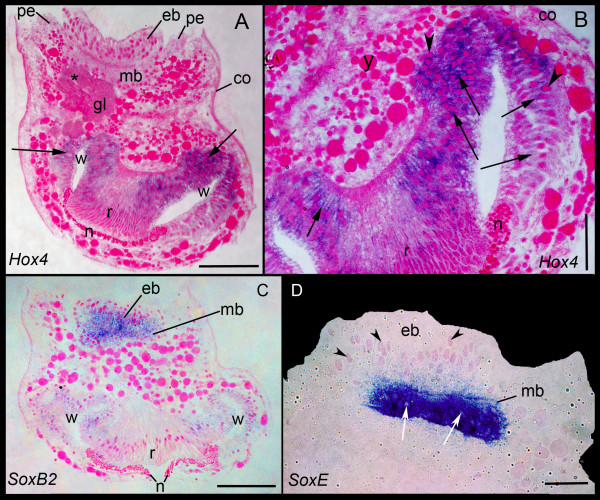
**Gene expression in semithin sectioned larvae**. (**A**) *BnHox4 *expression shown in lateral section and (**B**) magnified area of the abapical part of the larva showing the expression in the cell soma of the wall and roof cells of the internal sac. Expression in the cytoplasma of wall cells is indicated in some areas (arrows). Faint expression is present in the glandular area (gl) of the pyriform organ (asterisk). The nuclei of the wall cells are brightly stained with basic fuchsine (arrow heads). (**C**) *BnSoxB2 *expression is visible in the epidermal and mesodermal blastemas and wall cells. (**D**) Detail of the apical disc showing expression of *BnSoxE *in cells of the mesodermal blastema (two cells lacking expression are indicated by arrows). The epidermal blastema lacks expression (some basally lying nuclei of the cells of this layer are indicated by arrow heads). All sections are additionally stained with basic fuchsine (pink). co, corona; eb, epidermal blastema; pe, pallial epithelium; mb, mesodermal blastema; n, neck; r, roof; w, wall; y, yolk inclusion. Scale bars in (A, C) 50 μm; (B, D) 25 μm.

We observed *BnHox4 *expression in the wall and roof regions of the internal sac in the larvae (Figures 4A-C and 5A, B). Expression is also found internally in the glandular cell complex of the pyriform organ, as well as in the epidermal and mesodermal blastemas (Figure 4B).

#### SoxB2 and SoxE

*BnSoxB2 *expression is similar to *BnHox4 *expression (Figures 4D-F and 5C). In the bryozoan larva, *BnSoxE *is expressed in a subset of the *BnSoxB2 *expressing cells. The expression of *BnSoxE *is within two sickle shaped areas in the mesodermal blastema, which have their greatest extension in posterio-lateral position (Figures 4G-I and 5D). Additional faint expression is observed in the wall region of the internal sac (not shown).

#### BAMBI

Expression of *BnBAMBI *is found in a defined area of a trapezoid shape on the posterior side of the bryozoan larva, including cells of the epidermal and mesodermal blastemas, as well as some internal cells. In addition, cells positioned in the abapical wall region show expression, which gives a horseshoe-shaped appearance (Figure 4J-L).

#### FoxA, FoxB and FoxAB

**Figure 6:**
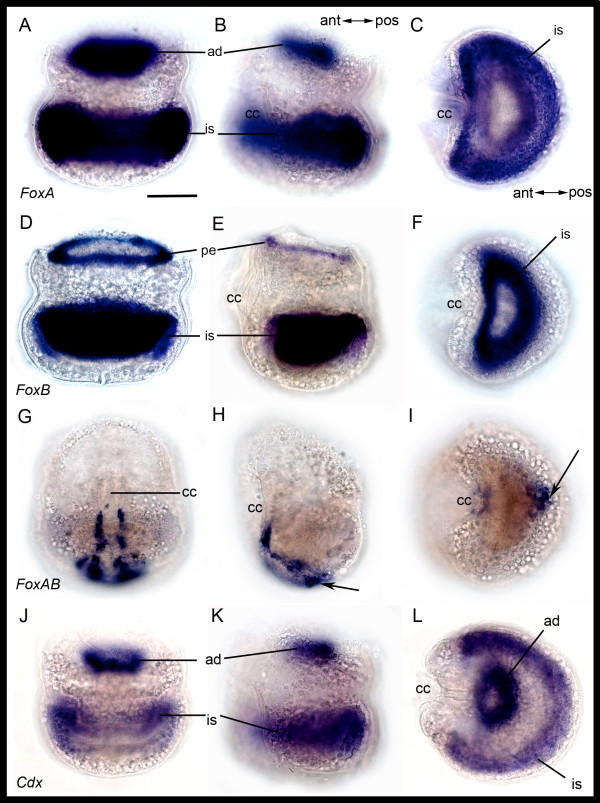
**Gene expression in *Bugula neritina *larvae**. Column 1. **A-D**, posterior view. J, anterior view. Column 2: Lateral view. Column 3: Abapical view. Row 1: Expression of *BnFoxA *in the epidermal and mesodermal blastema in the apical disc (ad) and the wall and roof region of the internal sac (is). Row 2: Expression of *BnFoxB *in the pallial epithelium (pe) and parts of internal sac. Row 3: Expression of *BnFoxAB *along the ciliated cleft (cc) and on the abapical side (arrow). Row 4: Gene expression of *BnCdx*, similar to *BnFoxA*. ant, anterior; pos, posterior. Scale bar 100 μm.

**Figure 7:**
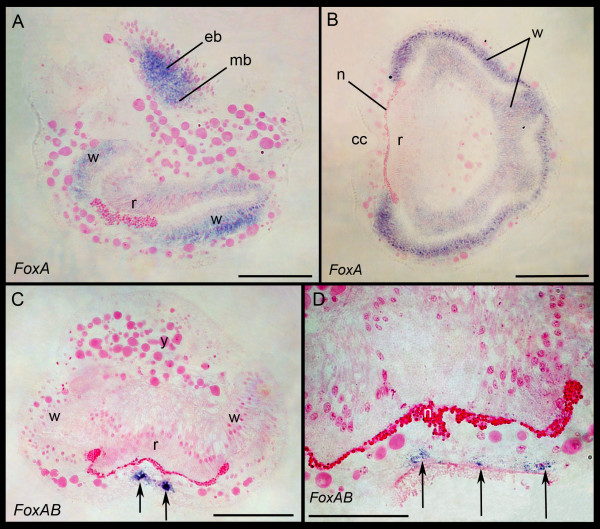
**Gene expression in semithin sectioned larvae**. (**A**) Lateral section showing *FoxA *expression in the apical disc and the wall (w) and roof (r) of the internal sac, similar to *SoxB2*. (**B**) Cross section showing *FoxA *expression in the wall of the folded internal sac. (**C**) Cross section in abapical area showing *FoxAB *expression in epidermal cells in the area of the ciliated cleft and (**D**) area around the ciliated cleft magnified with the expression indicated by arrows. All sections are additionally stained with basic fuchsine (pink). cc, ciliated cleft; eb, epidermal blastema; mb, mesodermal blastema; n; neck region of internal sac; y, yolk inclusion; Scale bars in (A, B, C) 50 μm; (D) 25 μm.

*BnFoxA *is expressed in a continuous ring in the epidermal and mesodermal blastemas, as well as in the wall and roof of the internal sac (Figures 6A-C and 7A, B). *BnFoxB *is co-expressed with *BnFoxA *in the wall cells of the internal sac, but also shows expression in the circular pallial epithelium (Figure 6D-F). *BnFoxAB *differs from the expression patterns of *BnFoxA *and *BnFoxAB *and is found in epidermal cells at the posterior side of the larvae along the edges of the ciliated groove, and the expression continues from anterior to posterior on the abapical side (Figures 6G-I and 7C, D).

#### Cdx (Caudal)

Similar to *BnFoxA, BnCdx *is expressed as a broad ring in the apical disc in cells of the epidermal and mesodermal blastemas, the wall region of the internal sac, and weak expression in the roof cells (Figure 6J-L).

#### GATA123 and GATA456

**Figure 8:**
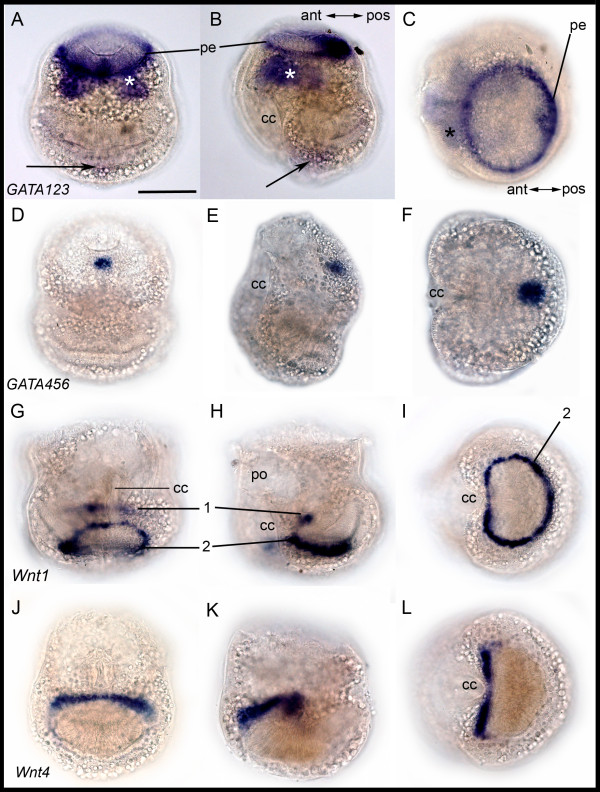
**Gene expression in *Bugula neritina *larvae**. Column 1: **A, D**: posterior view. **G, J**: anterior view. Column 2: **B**, **E**, **H**, **K**: lateral view. Column 3: **C, F**: apical view. **I, L**: abapical view. Row 1: *BnGATA123 *expression in the pallial epithelium (pe) and below it in the area of the pyriform organ (asterisk). Faint expression below the internal sac is indicated (arrows). Row 2: *BnGATA456 *expression in a single spot in posterio-apical position. Row 3: *BnWnt1 *expression pattern. 1 indicates posterior expression in some wall cells; 2 refers to the ring at abapical pole. Row 4: Expression of *BnWnt4 *in some wall cells. ant, anterior; cc, ciliated cleft; po, pyriform organ; pos, posterior.

**Figure 9:**
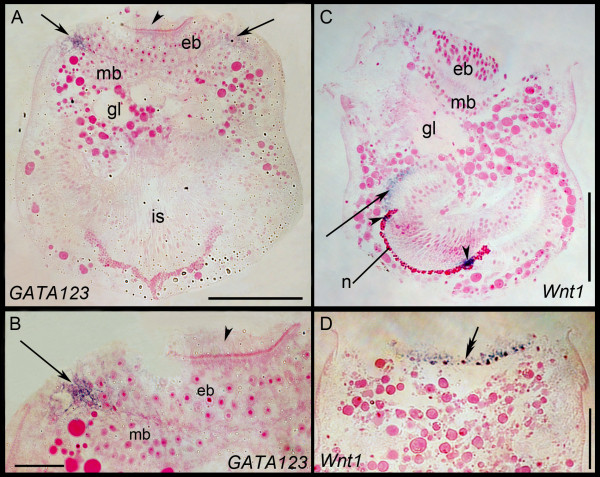
**Gene expression in semithin sectioned larvae**. (**A**) *BnGata123 *expression in the pallial epithelium (arrows) and area magnified in (**B**). (**C**) Lateral section of a larva showing the expression pattern of *BnWnt1 *in cells of the neck (arrowheads) and wall cells (arrow). The arrowhead points to cilia of the apical disc. (**D**) Expression of *BnWnt1 *in the pallial epithelium (double arrow). All sections are additionally stained with basic fuchsine (pink). eb, epidermal blastema; mb, mesodermal blastema; is, internal sac; gl; glandular area of the pyriform organ; n, granula in neck region; r, roof; w, wall; y, yolk droplet. Scale bars 100 μm in (A, C), 25 μm in (B, D).

The *BnGATA factor 123 *shows expression in a ring of cells in the pallial epithelium (Figures 8A-C and 9A, B). Expression is also observed in a few cells of the epidermal blastema and beneath the mesodermal blastema in the glandular area. Weak expression is present in the neck region of the internal sac (Figure 8A, B). In contrast, *BnGATA456 *is expressed in a single spot on the posterior side of the apical organ in the epidermal blastema of the *Bugula neritina *larvae (Figure 8D-F).

#### Wnt1 and Wnt4

*BnWnt1 *is expressed in cells of the neck region of the internal sac, appearing as a ring at the abapical pole. Further expression is found in cells of the internal sac apical to the neck cells and in the pallial epithelium (Figures 8G-I and 9C, D). *BnWnt4 *is co-expressed with *BnWnt1 *in some cells apical of the neck cells (Figure 8J-L). Additional expression is found in the abfrontal half of the pallial epithelium (not shown).

**Figure 10:**
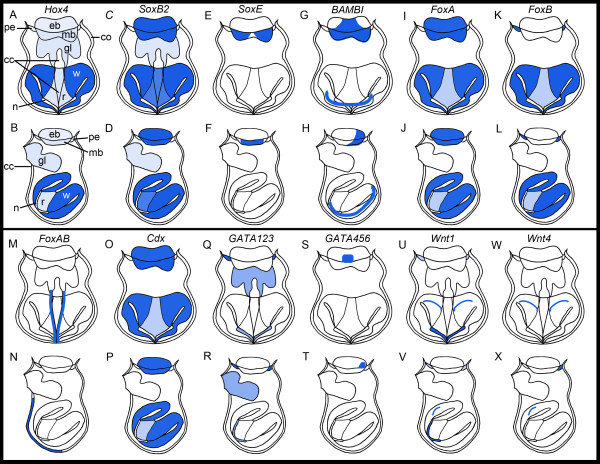
**Schematics of gene expression patterns of 12 genes in the coronate larva of *Bugula neritina***. Rows 1 and 3 represent larvae in sagittal section, in either an anterior angle (ciliated cleft = cc and glands of pyriform organ = gl indicated) or in a posterior angle (ciliated cleft not indicated). Rows 2 and 4 show larvae in lateral view with anterior side to the left. co, corona; eb, epidermal blastema; mb, mesodermal blastema; n, neck; pe, pallial epithelium; r, roof; w, wall.

A summary of all expression patterns (except *Tropomyosin*, see Figure 3) are presented schematically in Figure 10.

## Discussion

The lecitotrophic larva of *Bugula neritina *contains only a few truly larval organs, including the apical sense organ and associated nerves, the swimming organ (corona), the glandulo-sensory organ (pyriform organ), internal yolk cells, and muscles. These transitory larval structures are all likely to be discarded at metamorphosis [19]. Blastemic tissues that are known to form the adult during metamorphosis are the epidermal blastema, the mesodermal blastema, the pallial epithelium, and the internal sac. Of the 13 developmental genes that we investigated, 11 genes are expressed in discreet and highly reproducible regions of one or more of the blastemic tissues in the coronate larva that form the adult during metamorphosis. The results indicate that the blastemic cells are probably molecularly pre-determined in the bryozoan larva. Some of the 11 genes show additional expression in the pyriform organ, which is used by the larval stage and resorbed at metamorphosis. Only *BnTropomyosin *and *BnFoxAB *are solely expressed in transitory larval structures, the musculature and the pyriform organ (ciliated cleft), respectively (Figures 3, 10). In the following sections, we discuss the gene expression patterns of the bryozoan larval stage in respect to available data of other metazoans.

### Genes involved in neural development

#### Hox4, SoxB, SoxE, BAMBI and FoxB

In the animals investigated so far, *Hox *genes have been shown to play a crucial role in body plan regionalization along the anterior-posterior axis and are to a great extent expressed in ectodermal and mesodermal derivates. In Acoela, expression of a central *Hox *gene is found in ectodermal cells in early developmental stages which later give rise to putative neural precursor cells [26]. In several polychaetes, *Hox4 *is expressed in larval ectoderm and developing neural structures [27-29]. Accordingly, in the mollusk *Haliotis, Hox4 *expression is in presumptive neuroectoderm and developing ganglia in the trochophore stage and later in the mantle, suggesting partial co-option of the gene for shell formation [30]. Rather similar to *Hox4, SoxB *genes probably have a conserved role in nervous system development in cnidarians and spiralians [31-36]. In the bryozoan *Bugula, BnHox4 *and *BnSoxB2 *are widely co-expressed in both transitory larval structures as well as blastemic cells (Figure 10A, C). Ectodermal expression of *Hox4 *and *SoxB2 *in *Bugula *is similar to their expression in other metazoans, where they are involved in neuroectodermal patterning. Prior to our study, *SoxE *orthologs have only been investigated in deuterostomes, and in vertebrates they change the fate of neural stem cells into glial stem cells [37-39]. In the bryozoan, *BnSoxE *is expressed in a small subset of the *BnSoxB2 *expressing, blastemic cells (Figure 10E). *BAMBI *is an inhibitor of TGF-β signaling and expression was earlier observed in the beetle *Tribolium*, where it is co-expressed with *BMP/Dpp *in the dorsal ectoderm [40], and in deuterostomes, where *BAMBI *is co-expressed with *Bmp2/4 *orthologs [41-44]. *BnBAMBI *is expressed in a subset of the *BnSoxB2 *expressing cells, which form parts of the adult bryozoan (Figure 10G). *FoxB *orthologs play a role in neural development in the cnidarian *Nematostella*, the ecdysozoan *Drosophila*, and in chordates [33,45-48]. In *Bugula, BnFoxB *is exclusively expressed in future ectodermal tissues (Figure 10K).

The above considerations reveal that the bryozoan orthologs of *Hox4, SoxB2, SoxE, BAMBI*, and *FoxB *are to a great extent expressed in limited domains of the blastemas, and the expression patterns parallel that of other animal taxa to some degree. It is feasible that some of the investigated orthologs also play a role in nervous system patterning in bryozoans, and additional studies of gene function will be helpful to evaluate conserved and novel gene functions in the bryozoan life cycle.

### Genes involved in gut development

#### FoxA, Cdx and GATA456

The genes *FoxA, Cdx*, and *GATA456 *have previously been shown to be involved in bilaterian gut development. *FoxA *(*Forkhead, HNF3*) is important for the development of components of the digestive tract in protostomes as well as in deuterostomes [49-54]. In an acoel, *FoxA *is expressed in the endoderm surrounding the mouth, suggesting an ancestral role of *FoxA *in the endoderm and a later co-option in oral ectoderm in bilaterians [55]. In annelids, *FoxA *is expressed during gut formation [56-58], and in the mollusk *Patella, FoxA *expression is in the endoderm and in the developing foregut [59]. In the bryozoan larva, *BnFoxA *is expressed in different blastemas that give rise to adult tissues during metamorphosis (Figure 10I). Expression of *BnFoxA *in the epidermal blastema, which is supposed to form the adult digestive tract, suggests a function in gut development similar to other metazoans. *Cdx/Caudal *orthologs are involved in metazoan hindgut formation [60,61]. In addition, *Cdx *orthologs are expressed in the brains of both an acoel and an annelid [26,62]. In the mollusk *Patella, Cdx *expression is observed in the posterior neuroectoderm and mesodermal cells [63]. In the bryozoan larva, *BnCdx *is co-expressed with *BnFoxA *(Figure 10O), and it seems likely that *BnCdx *is partially involved in adult gut formation as in other lophotrochozoans. However, compared to other taxa, neither *FoxA *nor *Cdx *show positional pre-patterning in the bryozoan. The gene *GATA456 *is involved in endodermal specification in annelids [57,58]. In the bryozoan larva, *BnGATA456 *is co-expressed with *BnCdx *and *BnFoxA *in a confined domain in the epidermal blastema (Figure 10S).

Our results lead to the conclusion that the genes *FoxA, Cdx*, and *GATA456 *are probably involved in the formation of the adult digestive tract in bryozoans and other metazoans and that the epidermal blastema is pre-patterned according to its future fate. In this study, we find no sign of a vestigial larval gut in *Bugula neritina*, corroborating former histological investigations [22].

### Genes involved in ectoderm specification

#### GATA123

The *GATA123 *factor appears to be involved in ectodermal lineage specification in annelids [57,58,64], similar to what has been described for *Drosophila *[65]. We observed *BnGATA123 *expression to the greatest extent in ectodermal larval structures as well as in blastemic tissues that form the ectoderm of the adult (Figure 10Q), consistent with the ectodermal expression of the gene in other taxa.

### Genes for axial patterning

#### Wnt

*Wnt *signaling might be involved in the development of the primary body axis in most Metazoa [66]. However, from this suggested ancestral role, *Wnt *diversified and regulates many processes in metazoan taxa. A common feature of *Wnt *expression is a staggered arrangement along the anterior-posterior axis with partly overlapping domains in, for example, *Nematostella*, leech, or *Capitella *[67,68]. In *Bugula, Wnt1 *and *Wnt4 *expression is in specific, partly overlapping subsets of the future adult ectoderm, suggesting that the internal sac is regionalized and its cells might be differentially involved in adult body wall patterning (see also Figure 10U, W).

### Gene expression in transitory larval tissues

#### Tropomyosin and FoxAB

Most of the 11 genes mentioned above are largely expressed in subsets of progenitors of adult tissues, with only *BnTropomyosin *and *BnFoxAB *expressed exclusively in transitory larval tissues. *Tropomyosin *is a general marker of bilaterian musculature. The F-actin component of the larval musculature of *Bugula *species was investigated in previous studies and revealed prominent longitudinal and radial central muscles positioned between the internal sac and the apical organ, as well as body wall muscles [69,70]. The *BnTropomyosin *expression appears to conform with body wall muscles, and corresponds closely with a previous ultrastructural investigation of the *Bugula neritina *larva, which showed (1) the presence of myoepithelial cells between coronal cells, (2) a collarette of myoepithelial cells that join the infolded pallial epithelium, (3) a pair of myoepithelial cells extending along the lateral sides of the ciliated cleft and (4) a collarette joining the oral margin of the corona [21] (compare with Figure 3). The lack of *Tropomyosin *expression in blastemic tissues indicates that the adult musculature differentiates after metamorphosis.

Our study showed that *BnFoxAB *expression is confined to epidermal cells along the ciliated cleft and along a part of the abapical side of the bryozoan larva (Figure 10M). In a brachiopod larva, *FoxAB *expression was observed in cells of the apical pole instead (Hejnol *et al.*, unpublished data). Since no such expression is found in the bryozoan larva and comparable gene expression data of other metazoans are currently lacking, our conclusions must be provisional, but recruitment of *FoxAB *to larval structures may also be found in other taxa.

### Pluripotent stem cells versus pre-patterned adult tissues

It was earlier proposed that "homologous set-aside cells" exist in "homologous larvae of protostomes and deuterostomes" [71]. These set-aside cells were supposed to have a rather unlimited division capacity and remain pluripotent and undifferentiated until late embryogenesis. This idea is rooted in the hypothesis that early metazoans were similar to modern larvae, and that the bilaterian adult stage evolved by the innovation of set-aside cells, distinct from the larval cells [72]. However, this hypothesis would presume a similar gene regulatory system in all modern larvae. Here, we have shown that the blastemic cells ("set-aside cells") in the bryozoan larval stage express several metazoan developmental genes. The result indicates that the fate of the blastemic cells is already determined in the bryozoan larval stage and the blastemic cells of bryozoans are probably not pluripotent stem cells or set-aside cells. Our study rather suggests similarities between developmental gene expression in the bryozoan blastemas and adult tissues of other metazoans. In conclusion, this study does not support homology of the bryozoan larval stage and other lophotrochozoan larvae. Instead, the gene expression patterns presented herein indicate that planktonic larvae might have secondarily evolved in bryozoans. The latter hypothesis needs to be evaluated by further studies of gene expression and gene function during the development of bryozoans as well as other lophotrochozoans.

## Conclusions

We have shown here the gene expression of 13 developmental genes (*Tropomyosin, Hox4, SoxB2, SoxE, BAMBI, Cdx, FoxA, FoxB, FoxAB, GATA123, GATA456, Wnt1*, and *Wnt4*) in the coronate larva of the bryozoan *Bugula neritina*. Eleven of the 13 genes are expressed in blastemic cells, which are precursors of adult tissues. Probably only a few of the investigated genes have their main function in the larval stage, as for example, *BnTropomyosin*, which is expressed in larval muscle cells. This study reveals that the blastemic cells in the bryozoan larval stage are most probably pre-patterned according to their future fate in the adult and are not pluripotent, undifferentiated set-aside cells as previously stated [71]. Our results contradict the idea that larval and adult bodies are different entities, but that there is a natural transition from the embryo to the adult with an intermediate larval stage. A comparison of expression patterns among metazoans reveals molecular similarities between bryozoan blastemas and adult tissues of other metazoans. Hence, our study does not indicate homology of the bryozoan larva with other lophotrochozoan larvae, but instead suggests conserved patterns of developmental gene expression amongst lophotrochozoan and metazoan adults. This study adds important data to the fundamental discussion about the evolution of metazoan larval stages and should trigger the interest in investigating gene expression in the "set-aside cells" of the larval stage of other lophotrochozoans as, for example nemerteans. Cell-lineage studies of bryozoans and additional gene expression studies during bryozoan development will also contribute to our understanding of the evolution of metazoan life cycles.

## Methods

### Collection of bryozoans

Colonies of *Bugula neritina *were collected from submerged hard substrates in shallow water depth (0 to 3 m) in harbors of Honolulu ("La Mariana" and Kewalo Basin) and Pearl Harbor, Oahu, Hawaii in May and June 2009. The colonies were kept in flowing seawater tables in the dark at the Kewalo Marine Laboratory (Hawaii) for a minimum of one day. By exposing colonies to pointed light sources, larvae were released from spawning colonies. They were immediately collected from the water surface under dissecting microscopes and prepared for further investigation.

### RNA isolation and cDNA synthesis

Larvae were fixed and stored in RNA*later *at 4°C. Larval mRNA was obtained using DynaBeads mRNA DIRECT Kit (Invitrogen, Carlsbad, CA, USA) according to the supplier and stored at -80°C. Complementary DNA (cDNA) synthesis was achieved using the Advantage RT-for-PCR Kit protocol (Clontech Laboratories, Mountain View, CA, USA) following the supplier's instructions. cDNA was stored at -20°C.

### Gene isolation

The sequences for the genes *BnTropomyosin, BnBAMBI, BnSOXB2, BnSoxE BnHox4, BnFoxB, BnFoxAB, WNT1 *were gained from a public EST library of *Bugula neritin*a [3]. Fragments for *BnCdx, BnFoxA, BnWNT4 *and both *GATA *factors were gained using degenerate primers with larval cDNA as template. Sequences of the genes from the EST library were amplified using gene specific primers and degenerate fragments were extended using rapid amplification of cDNA ends (RACE) with a SMART RACE cDNA amplification kit (Clontech Laboratories, Mountain View, CA, USA). All fragments were cloned into pGEM-T Easy vectors (Promega Corporation, Madison, WI, USA), transformed into *E.coli*, and clones sequenced at Macrogen, Inc. (Seoul, South Korea). Fragments obtained from *B. neritina *were used for probe synthesis in *in situ *hybridization reactions, which are described below. Primer sequences are listed in Additional file 1 Table S1. Genes were deposited at NCBI GenBank (see below).

### Gene accession numbers

*BnBAMBI *[GenBank: HQ914790]; *BnCdx *[GenBank: HQ914791]; *BnFoxA *[GenBank: HQ914792]; *BnFoxAB *[GenBank: HQ914793]; *BnFoxB *[GenBank: HQ914794]; *BnGATA123 *[GenBank: HQ914795]; *BnGATA456 *[GenBank: HQ914796]; *BnGsc *[GenBank: HQ914797]; *BnHox4 *[GenBank: HQ914798]; *BnSoxB2 *[GenBank: HQ914799]; *BnSoxE *[GenBank: HQ914800]; *BnTropomyosin *[GenBank: HQ914801]; *BnWnt1 *[GenBank: HQ914802]; *BnWnt4 *[GenBank: HQ914803]; *BnWnt8 *[GenBank: HQ914804].

### Gene orthology assignment

Gene orthologies for all genes (except for *BnBAMBI *and *BnTropomyosin *of which the orthology was detected by alignments) were determined by phylogenetic analyses using PhyML 3.0 [73]. Alignments were conducted using MUSCLE [74] and corrected by hand. ProtTest [75] was used to determine the best-fitting model. A total of 1,000 to 3,000 bootstraps were calculated respectively, see Additional data files.

### *In situ *hybridization protocol

Larvae of *B. neritina *were relaxed in 7.14% MgCl_2_, prefixed in glutaraldehyde fixative (0.3% glutaraldehyde, 3.7% formaldehyde in seawater) for two minutes, fixed in 3.7% formaldehyde for one hour at 4°C, washed five times in PTw (1x PBS = phosphate buffered saline + 0.1% Tween 20) and once in distilled water, which was replaced three times by 100% methanol. The larvae were finally stored in the latter at -20°C. All further steps follow the protocol of Hejnol *et al. *[55]. Following the development of the *in situ *probes, larvae were then transferred into 70% glycerol and whole-mounted on glass slides and expression patterns were imaged with a Nikon DXM1200 digital camera mounted on a Nikon Eclipse E1000 microscope. For detailed examination of the expression patterns, larvae were also prepared for histology.

### Histology of larva after *in situ *hybridization

For the histological preparations, *in situ *hybridized larvae stored in 70% glycerol were washed three times in PBS and dehydrated in an ascending ethanol series with a final step of dehydration in 100% propylene oxide. The larvae were transferred into a 1:1 mixture of 100% propylene oxide and Low Viscosity Resin (LVR) (Agar Scientific, Stansted, UK) over night for infiltration. The animals were embedded in 100% LVR and semithin serial sections (2 μm) were performed on a Leica RM2255 microtome using a Diatome Histo Jumbo Diamond Knife (Diatome, Hatfield, PA, USA). Sections were stained with a 1% solution of basic fuchsine (p-Rosanilin) in 70% ethanol or toluidine blue and embedded in LVR on slides. Imaging was performed with the Nikon setup described in the previous section.

## Abbreviations

Bn: *Bugula neritina*; cDNA: complementary DNA; DNA: deoxyribonucleic acid; EST: expressed sequence tag; mRNA: messenger ribonucleic acid; PBS: phosphate buffered saline; PCR: polymerase chain reaction; LVR: low viscosity resin; RACE: rapid amplification of cDNA ends

## Competing interests

The authors declare that they have no competing interests.

## Authors' contributions

JF and AH performed the molecular laboratory work and JF conducted histology and imaging. AH coordinated the project and performed the phylogenetic analyses. JF wrote the draft manuscript and AH and MQM participated in manuscript preparation. All authors read and approved the final version of the manuscript.

## Supplementary Material

Additional file 1 **Primer sequences and gene orthology analyses**. Degenerate primer sequences (Table S1) and trees of the orthology analyses of the *Bugula neritina *genes *BnSoxB2, BnSoxE, BnFoxA, BnFoxB, BnFoxAB, BnHox4, BnCdx, BnGATA123, BnGATA456, BnWnt1, BnWnt4*, and *BnWnt8 *(Figures S1 - S5). Click here for file
