# Supplementary material for: Gene expression in bryozoan larvae suggest a fundamental importance of pre-patterned blastemic cells in the bryozoan life-cycle
Source: EvoDevo. 2011 Jun 6;2:13. doi: 10.1186/2041-9139-2-13 (PMC3133996; doi:10.1186/2041-9139-2-13)
Supplement: Additional file 1 — Primer sequences and gene orthology analyses. Degenerate primer sequences (Table S1) and trees of the orthology analyses of the Bugula neritina genes BnSoxB2, BnSoxE, BnFoxA, BnFoxB, BnFoxAB, BnHox4, BnCdx, BnGATA123, BnGATA456, BnWnt1, BnWnt4, and BnWnt8 (Figures S1 - S5). [file 2041-9139-2-13-S1.PDF]

Additional Files

Table 1: Degenerate Primer Sequences

Gene	Primer F1	Primer F2	Primer R1	Primer R2
**Cdx/caudal**	5’ – AARACNMGNACNAARGAYAARTA – 3’	5’ – GAYAARTAYMGNGTNGTNTA – 3’	5’ – TTNCKYTCYTTNGCNCKNCKRTT – 3’	5’ – TTNGCNCKNCKRTTYTGRAACCA – 3’
**forkhead**	5’ – AARCCNCCNTAYWSNTAYAT – 3’	5’ – TAYATHWSNYTNATHACNATG – 3’	5’ – TARCANCCRTTYTCRAACAT – 3’	5’ – CCRTTYTCRAACATRTTNCC – 3’
**GATA**	5’ – GARTGYGTNAAYTGYGGNGC – 3’	5’ – GGNCAYTAYYTNTGYAAYGC – 3’	5’ – GGYTTNCKYTTNCKNGTYTG – 3’	5’ – GTYTGDATNCCYTCYTTYTTCAT – 3’
**WNT**	5’ – ATGAAYYTICAYAAYAAYGA – 3’	5’ – TGYAARTGYCAYGGIGTIWSIGG – 3’	5’ – RCARCACCARTGRAAITTRCA – 3’	

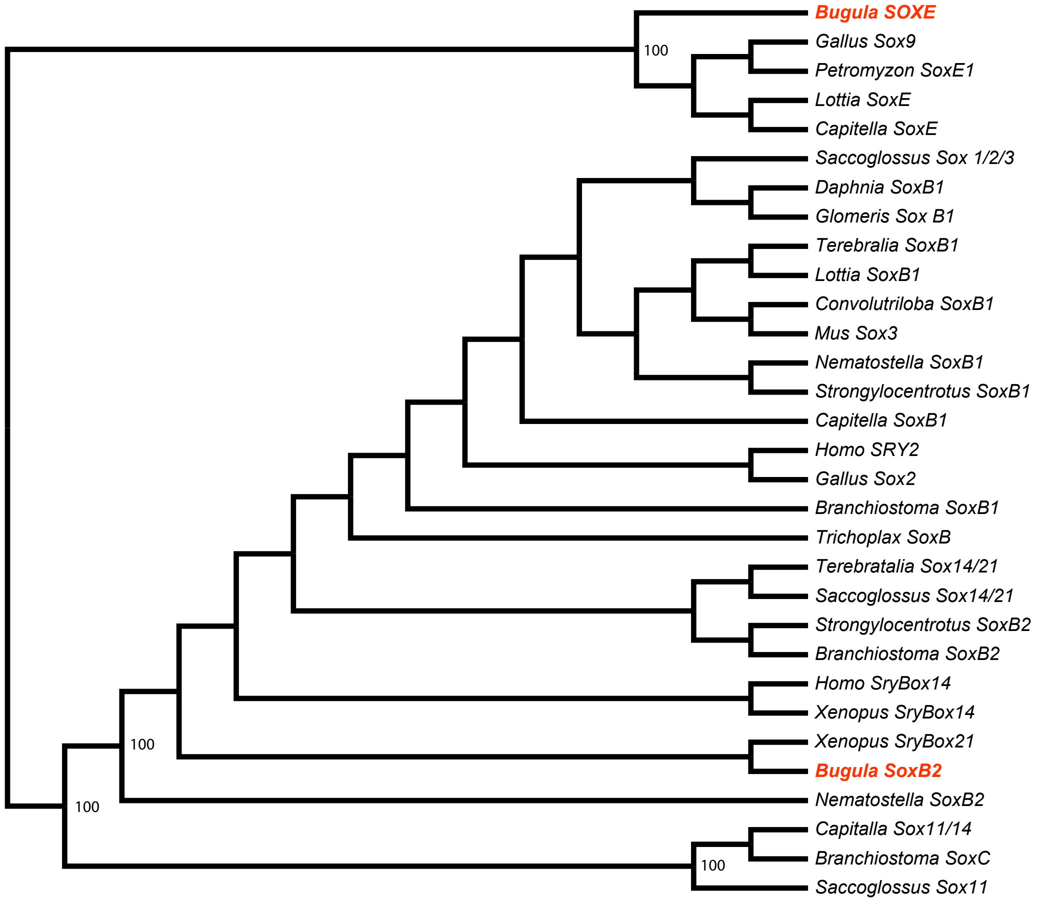

Figure 1: Maximum Likelihood phylogenetic orthology assignment of *Sox* genes. (3000 bootstrap replicates)

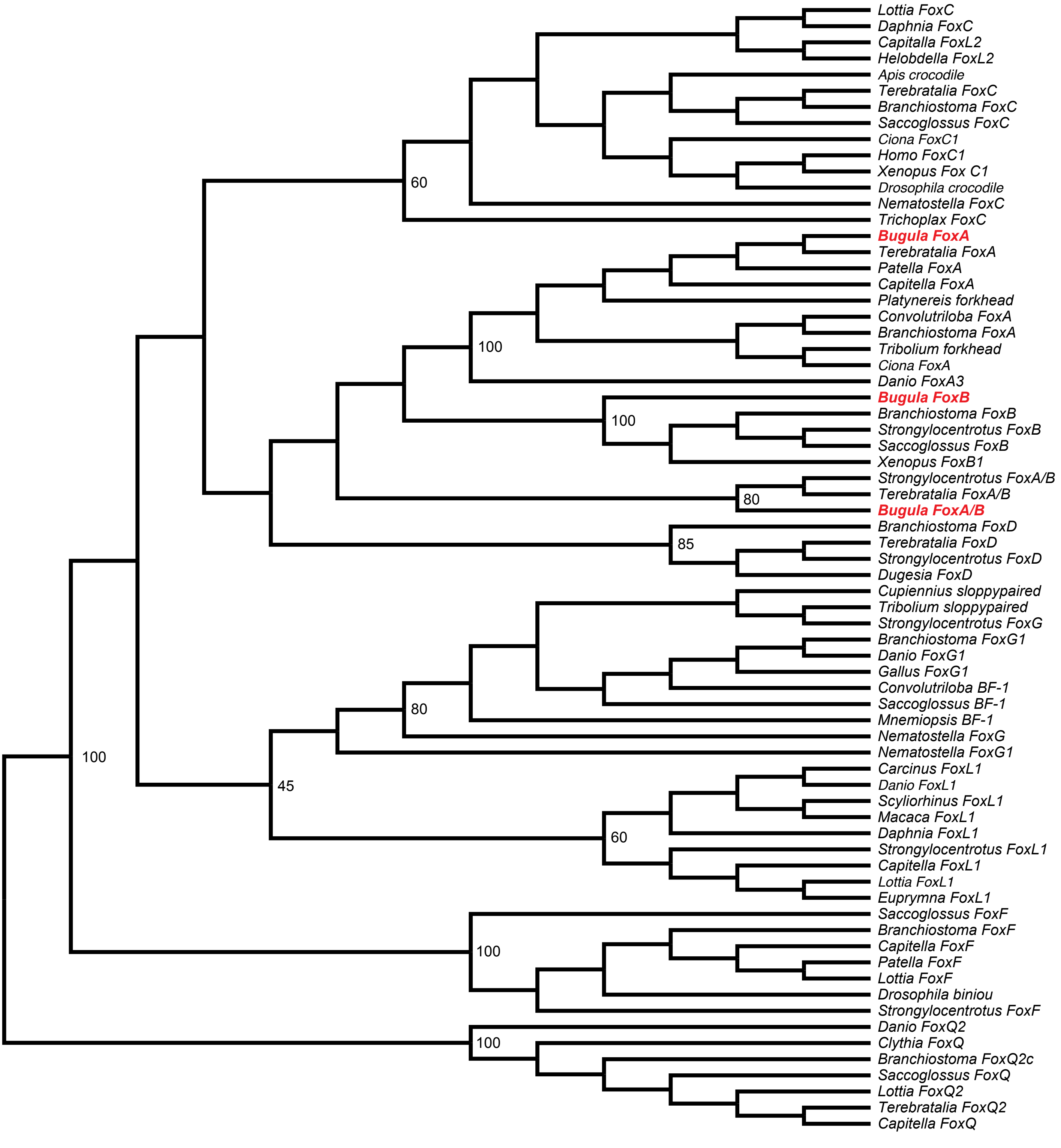

Figure 2: Maximum Likelihood phylogenetic orthology assignment of *Fox* genes. (2000 bootstrap replicates)

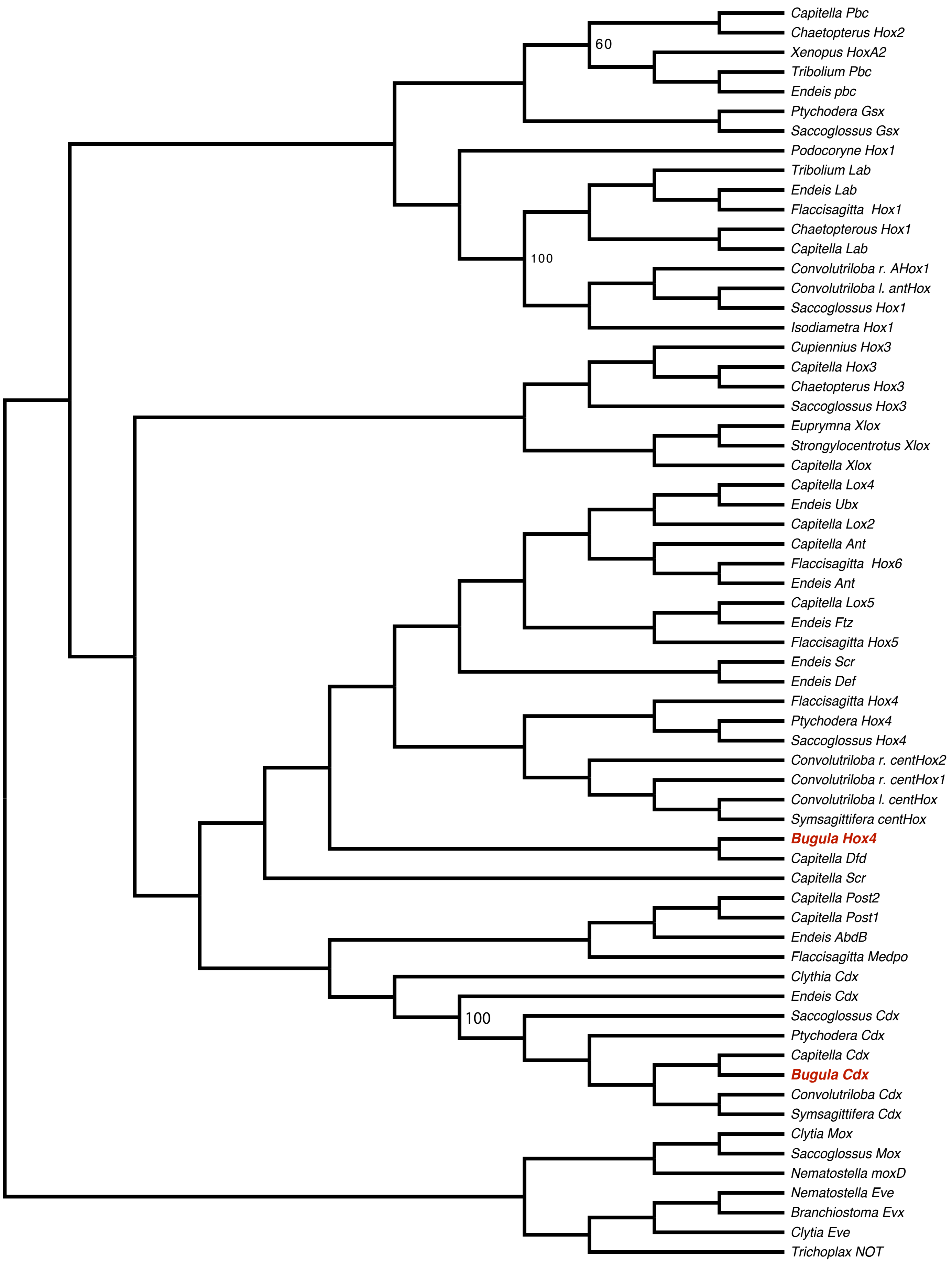

Figure 3: Maximum Likelihood phylogenetic orthology assignment of *Hox/ParaHox* genes. (2000 bootstrap replicates)

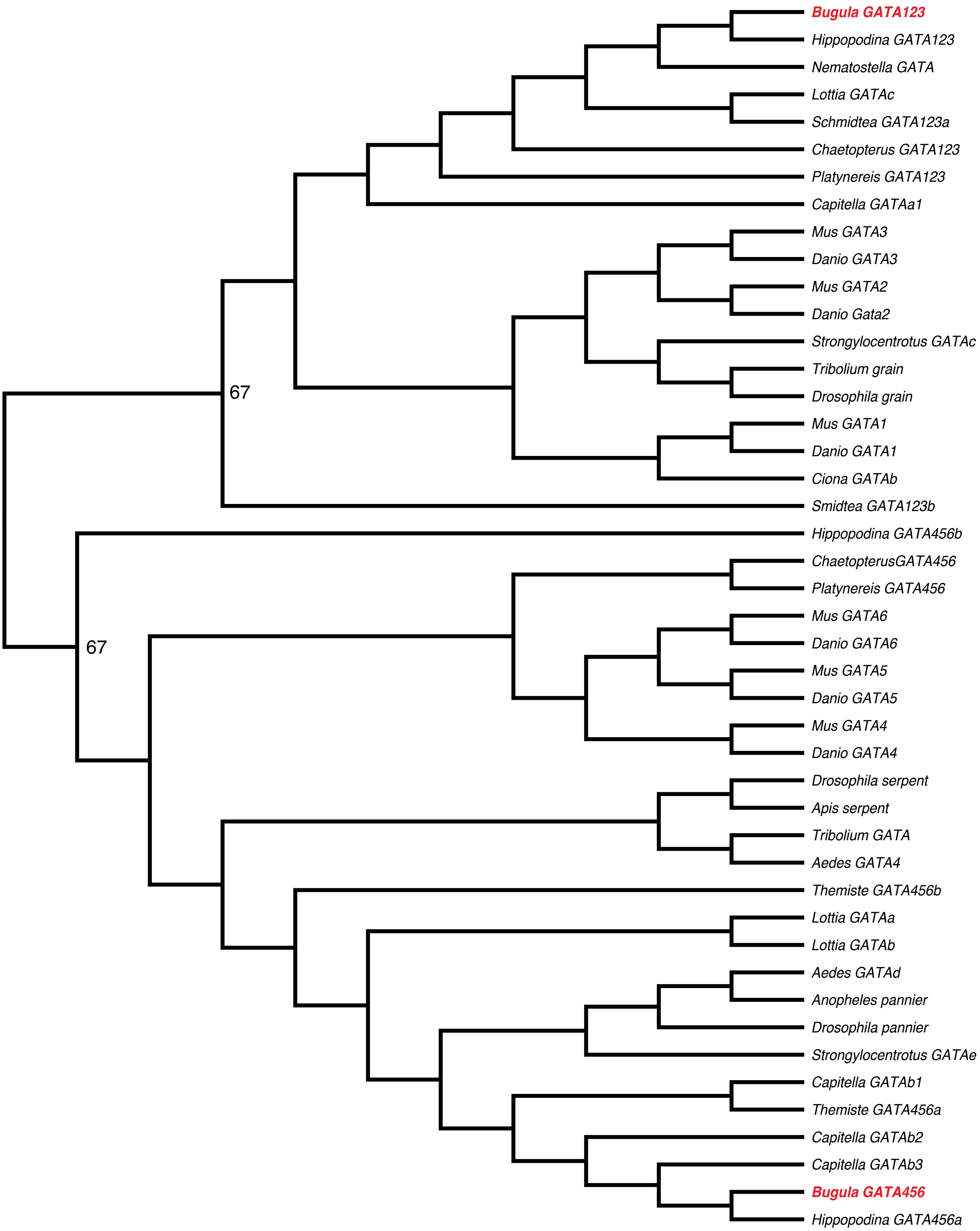

Figure 4: Maximum Likelihood phylogenetic orthology assignment of *GATA* genes. (3000 bootstrap replicates)

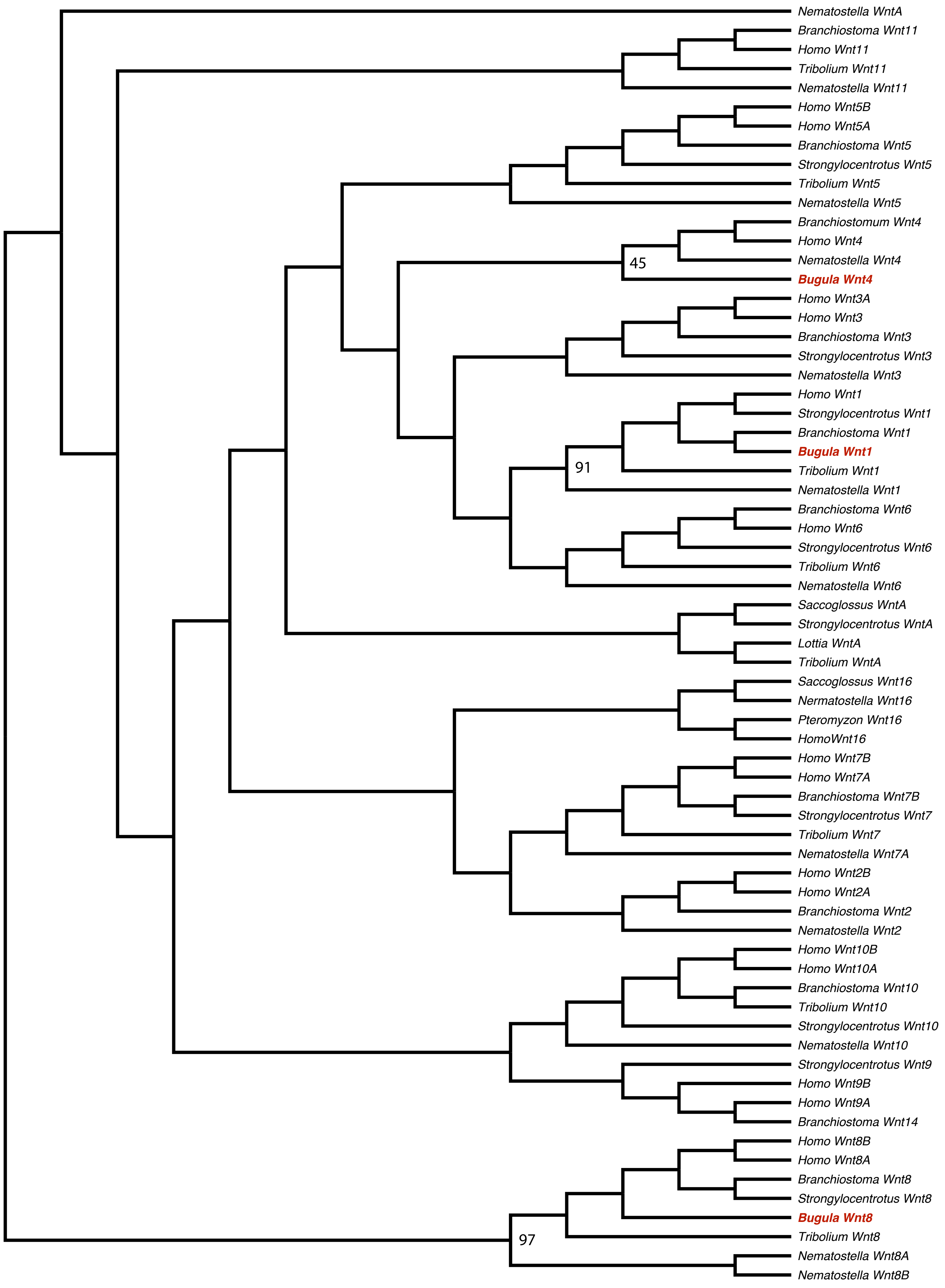

Figure 5: Maximum Likelihood phylogenetic orthology assignment of *WNT* genes. (1000 bootstrap replicates)
